# Telomerase RNA evolution: a journey from plant telomeres to broader eukaryotic diversity

**DOI:** 10.1042/BCJ20240501

**Published:** 2025-01-31

**Authors:** Petr Fajkus, Jiří Fajkus

**Affiliations:** 1 Institute of Biophysics of the Czech Academy of Sciences, 612 65 Brno, Czech Republic; 2 Laboratory of Functional Genomics and Proteomics, National Centre for Biomolecular Research, Faculty of Science, Masaryk University, 625 00 Brno, Czech Republic; 3 Mendel Centre for Plant Genomics and Proteomics, CEITEC - Central European Institute of Technology, Masaryk University, 625 00 Brno, Czech Republic

**Keywords:** non-coding RNA, telomerase RNA, telomere, TR evolution, TR identification

## Abstract

Telomeres, essential for maintaining genomic stability, are typically preserved through the action of telomerase, a ribonucleoprotein complex that synthesizes telomeric DNA. One of its two core components, telomerase RNA (TR), serves as the template for this synthesis, and its evolution across different species is both complex and diverse. This review discusses recent advancements in understanding TR evolution, with a focus on plants (Viridiplantae). Utilizing novel bioinformatic tools and accumulating genomic and transcriptomic data, combined with corresponding experimental validation, researchers have begun to unravel the intricate pathways of TR evolution and telomere maintenance mechanisms. Contrary to previous beliefs, a monophyletic origin of TR has been demonstrated first in land plants and subsequently across the broader phylogenetic megagroup Diaphoretickes. Conversely, the discovery of plant-type TRs in insects challenges assumptions about the monophyletic origin of TRs in animals, suggesting evolutionary innovations coinciding with arthropod divergence. The review also highlights key challenges in TR identification and provides examples of how these have been addressed. Overall, this work underscores the importance of expanding beyond model organisms to comprehend the full complexity of telomerase evolution, with potential applications in agriculture and biotechnology.

## Introduction

Telomeres, the protective caps at the ends of linear chromosomes, are essential for maintaining genomic stability in eukaryotic cells. In most organisms, these structures are composed of repetitive DNA sequences bound by specific proteins, which prevent chromosome ends from being recognized as DNA damage sites. However, due to the end-replication problem—where DNA polymerases cannot fully replicate the 3′ ends of linear chromosomes [1,2]—telomeres progressively shorten with each cell division. To counteract this, many eukaryotes, including plants, employ the enzyme complex telomerase, which extends telomeres by adding telomeric repeats to the chromosome ends [3-5].

The ribonucleoprotein (RNP) complex of telomerase is composed of two core components: the telomerase reverse transcriptase (TERT) protein and the telomerase RNA (TR). The TR component is a non-coding RNA (ncRNA) that serves as a template for the synthesis of telomeric DNA by TERT and is thus crucial for the enzymatic activity of telomerase [4]. Additionally, TR functions as a scaffold, facilitating the assembly of the telomerase RNP complex, which is essential for telomere maintenance [6-9]. While the overall function of TR is conserved across eukaryotes, the structure, sequence, and biogenesis of this RNA vary significantly between different kingdoms of life, reflecting the evolutionary divergence among these groups.

Although the first telomere DNA in any higher eukaryote was first characterized in *Arabidopsis thaliana* [10], subsequent telomere biology research has primarily focused on humans and model yeasts. This focus is understandable from the point of view of potential applications of telomere biology in cancer therapy or regenerative medicine. However, limiting research to such models restricts the discovery of novel mechanisms involved in telomere synthesis and the evolutionary complexity of telomeres, telomerase, and their components.

### Telomeres and telomerase in plants

Our interest in plant telomere biology was motivated by several factors. Initial studies demonstrated that, in contrast with other organisms, plant telomerase is inducible and reversibly regulated based on the proliferative activity of the cells [5,11-13]. Because telomerase remains active in dividing plant tissues (meristems) throughout the plant’s development, there is no progressive telomere shortening, unlike in most human somatic cells. Notably, telomerase activity can also be reactivated in differentiated cells and tissues when cell proliferation is induced, such as during regeneration [12]. A central aim of telomerase research in plants remains to elucidate the molecular basis of this reversible regulation.

Another reason for studying telomeres and telomerase in plants is the unusually high evolutionary variability of plant telomeres [14-19]. Given that the sequence of the TR template region determines the synthesis of telomeric DNA, research into the molecular causes of telomere variability led to the identification of corresponding TRs. A key follow-up question was how such changes in TR and telomere structure occur, considering the risks posed by alterations in telomeric DNA, which could destabilize interactions with telomere-binding proteins that recruit telomerase to chromosome ends.

Beyond academic curiosity, the study of plant TR and telomere biology has also practical applications, particularly in the targeted modification of chromosome stability in agriculturally important crops. This problem may be particularly important in allopolyploid crops, where multiple TR genes and other components of the telomere maintenance machinery are encountered, and interference may occur between them (see below).

### Structure, function, and identification of plant telomerase RNA

The catalytic subunit of telomerase, telomerase reverse transcriptase (TERT), is relatively conserved across eukaryotes (except for the N-terminal domain), showing evolutionary relatedness to reverse transcriptase of Penelope-like retroviruses [20,21]. Consequently, its bioinformatic identification is relatively straightforward as we have recently demonstrated [22]. In contrast, telomerase RNAs (TRs) are extremely diverse in terms of size, sequence, structure, and their ncRNA biogenesis pathways (reviewed in [23]), making TR identification and characterization highly challenging.

Biochemical approaches that successfully identified TRs in *Tetrahymena* and other ciliates, where TRs are highly abundant [24-26], appeared problematic in most other eukaryotes. In the yeast *Saccharomyces cerevisiae*, TR (referred to as telomerase component 1, TLC1) was discovered through a genetic screen for suppressors of telomeric silencing [27]. A different strategy was used to characterize human TR (hTR), which has much lower cellular abundance (hundreds of copies versus approximately 20,000 in *Tetrahymena* [28]). This approach involved subtractive hybridization using cDNA libraries from telomerase-positive and telomerase-negative cell lines. In addition, an independent cDNA library, enriched for potential TRs, was prepared from partially purified telomerase enzyme fractions. The resulting telomerase-enriched cDNA libraries were further screened for template-containing sequences by hybridization to a biotinylated 12-nucleotide complement of the predicted template, and this process was repeated 3–4 times [29]. Concurrently, mouse TR (mTR) was cloned from a mouse genomic lambda library using hTR as a probe and was found to be only 58% identical to hTR [30]. These pioneering studies demonstrated the aforementioned diversity of TRs. While ciliates have short ncRNA TRs (less than 200 nucleotides in length) transcribed by RNA polymerase III, *S. cerevisiae* TR/TLC1 is a 1.3 kb-long non-coding RNA (lncRNA) transcribed by RNA polymerase II, and human and mouse TRs are H/ACA box small nucleolar RNAs (snoRNAs) transcribed by RNA polymerase II, 451 and 397 nucleotides long, respectively.

The identification of TR in plants has been somewhat complicated. Using a biochemical approach, the first TRs in *A. thaliana* were reported as two alternative TR components (referred to as TER1 and TER2), associating with *Arabidopsis* POT1a and POT1b shelterin homologs, respectively [31,32]. These TRs were transcribed by RNA polymerase II and did not show homologs even in closely related species. The authors explained this surprising finding by proposing a polyphyletic nature of plant TRs and the flexible selection of suitable lncRNAs for the templating function by plant TERT [33].

Several years later, we combined transcriptomics and bioinformatics to identify TRs in the plant genus *Allium* and related Asparagales plants. Our aim was to find a mechanistic explanation for the previously described evolutionary changes of telomeres in the Asparagales phylogeny—specifically, the switch from *Arabidopsis*-type TTTAGGG telomere repeats to human-type TTAGGG repeats in the divergence of the Iridaceae family [16] and subsequently to the unusually long 12-nt repeats TTATGGGCTCGG in the *Allium* genus [19].

The unusually long telomere repeat unit in *Allium* allowed us to design an original approach to search for the minimal template region of the putative TR. By definition, the minimal template region must be at least one nucleotide longer than the length of the synthesized telomere repeat. Therefore, we searched for 13-nt circular permutations of the *Allium* telomere repeat. Putative TRs were searched in whole transcriptomes (partially depleted of rRNA) of six *Allium* species. Only 1–7 candidate transcripts were identified in individual species, sharing 85% pairwise identity compared with the consensus sequence, suggesting that they represented orthologs of the *Allium* TR. We extended this approach to species with TTAGGG repeats, including *Tulbaghia violacea* and *Scilla peruviana* [16,34]. Subsets of candidate TRs were analyzed using the *Allium cepa* TR (AcTR) sequence as a query in a BLASTN search. In parallel, we searched for putative orthologs of AcTR in publicly available genomic and transcriptomic data from various species of the order Asparagales and cross-mapped candidate hits back to our datasets. We identified six TR candidates in different Asparagales plant families that share the human-type telomeric repeat, i.e. Amaryllidaceae (*T. violacea*, *Rhodophiala pratensis*) and Asparagaceae (*S. peruviana*, *Agave tequilana*, *Nolina bigelovii*, and *Asparagus officinalis*) [35]. As a proof of concept, we also identified putative TR orthologs from *Dendrobium catenatum* and *Phalaenopsis equestris* (Orchidaceae), which are members of plant families in Asparagales sharing an *Arabidopsis*-type telomeric repeat [16].

Comparison of putative TRs from three plant groups in Asparagales clearly showed that the sequence of the template region corresponds to the expected type of telomeric repeat—either *Arabidopsis*, human, or onion type—synthesized by the respective telomerases [35]. These results indicated that the origin of plant TRs could be monophyletic. Therefore, we searched for corresponding TR orthologs in available datasets from representative monocot, eudicot, and gymnosperm species. This approach allowed us to identify candidate TRs in datasets from representative species across the land plant phylogeny.

Comparison of these candidate TR sequences revealed conserved regions within the transcribed parts that are shared among plant TRs and may be important for TR secondary structure: a 5′-G-rich region, the template domain, a conserved region downstream from the template domain, a C-rich region, and an AT-rich region near the 3′ end. Comparison of TR candidates found within genomic datasets enabled further identification of conserved regions of plant TR genes with putative regulatory functions, i.e. a TATA box, an upstream sequence element (USE), and a terminator. Subsequent analysis of plant TR secondary structure [36] revealed structural elements resembling ciliate TRs and vertebrate TRs. The TR core comprises the pseudoknot domain (P2 and P3 stems), template region, and the template boundary element (TBE), which varies across plant lineages. In gymnosperms and lycophytes, the TBE is formed by consecutive P1.1a and P1.1b stems, while in angiosperms, its function in template definition is supplemented by the central P1c stem. Beyond the TR core, additional conserved structural elements—P4/P5/P6 consecutive stems and P1 closing stem—contribute to telomerase assembly and function.

A surprising result came from the model species *A. thaliana*, in which we found an orthologous TR sequence corresponding to the lncRNA gene R8, previously described for its functions in hypoxic stress and RNA polymerase III transcription [37]. This candidate TR, different from the previously described TER1 and TER2, contains the sequence CTAAACCCT within the putative template region, clearly defining an *Arabidopsis*-type telomeric repeat with an additional two nucleotides that can serve as an anchor site for telomerase annealing to telomeres. TRs with corresponding template regions were also found in previously identified plant species with unusual telomere repeats, *Genlisea hispidula* (TTCAGG and TTCAGGG) [17] and *Cestrum elegans* (TTTTTTAGGG) [15,18]. Together, these results confirmed that changes in the telomere motifs synthesized are dictated by sequence variation within and around the central motif ACCCTAA [35].

Functionality of TRs identified in *A. cepa* and *A. thaliana* was then verified using telomerase activity reconstitution experiments, as well as telomere and telomerase analysis in TR knockout mutants and their complementation. Simultaneously, we demonstrated the lack of telomerase-related function in the previously misidentified TER1 and TER2 [31]. The newly identified *A. thaliana* TR was also supported in independent studies using TR enrichment through an antibody against the TERT subunit [36,38]. These results ultimately changed earlier views on genuine plant TRs: they are of monophyletic origin and are transcribed by RNA polymerase III under the control of a type-3 promoter, which consists of a specifically spaced TATA box and a USE [39-41]. In addition to the identified TRs, our approach makes it possible not only to identify unknown TRs in land plants as soon as their genomic or transcriptomic data are available but also to directly predict telomere sequences based on the template regions of these TRs.

### Common origin of plant TRs predates the emergence of land plants

**Figure 1:**
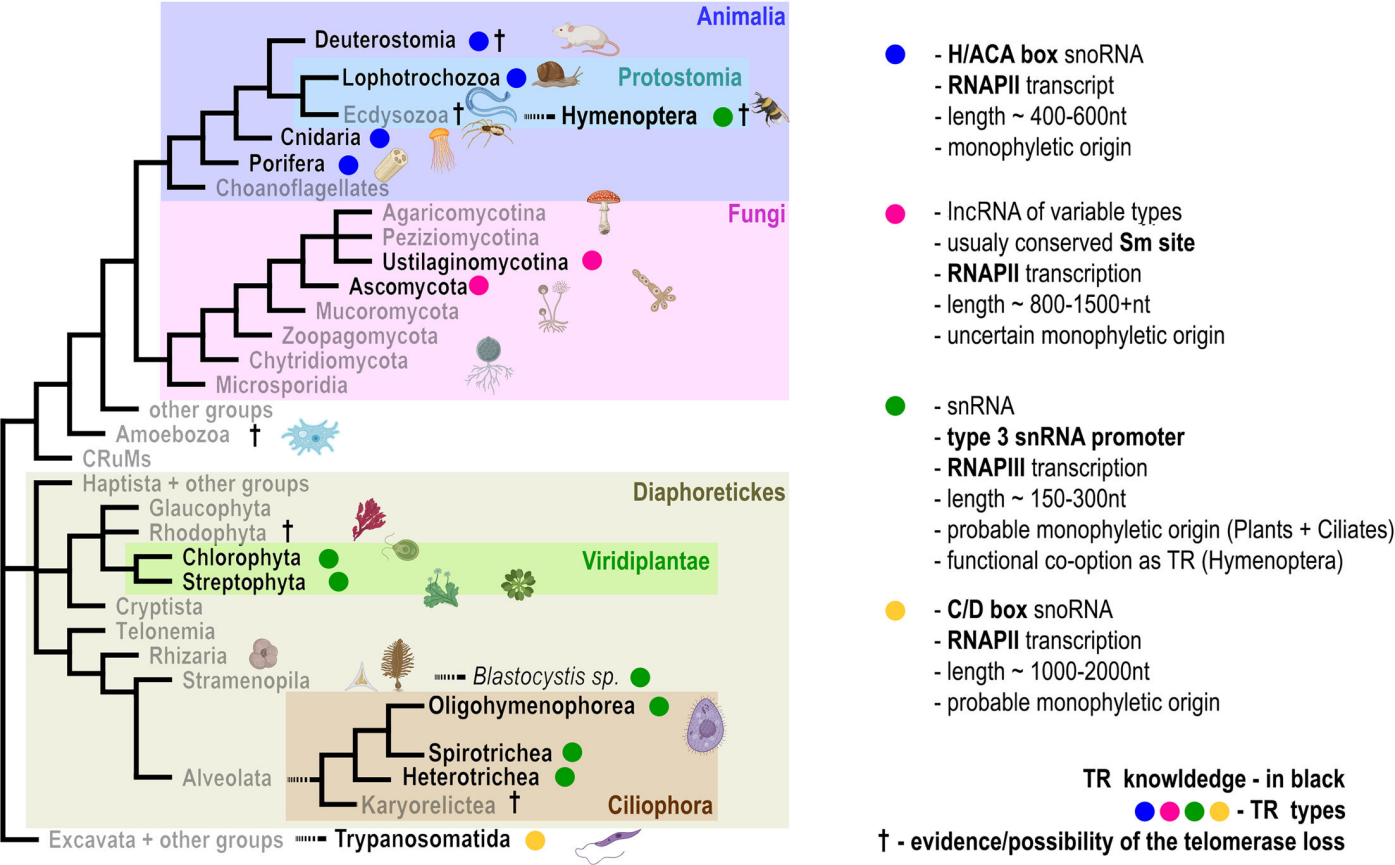
Phylogenetic tree of eukaryotes highlighting taxa with known telomerase RNA (TR) characteristics (indicated in black). TR types are represented by colored dots, reflecting the diversity in RNA features across taxa as detailed in the right panel. The figure emphasizes the switch in RNA type observed in Hymenoptera, transitioning from the H/ACA box snoRNA characteristic of Animalia to TR types typical of ciliates and plants. Understanding unknown TR characteristics in Ecdysozoa (e.g. nematodes and arthropods) would substantially enhance our knowledge of the evolutionary dynamics and functional diversity of telomerase RNA across eukaryotic lineages. The † symbols indicate evidence or possibilities of telomerase loss in specific groups. Besides well-studied diptera/*Drosophila*, potential loss of telomerase has been reported in amphibian *Pleurodeles waltl* (Deuterostomia) [42], Cynipoidea (Hymenoptera) [22], spiders (Araneae) [43], genus *Meloidogyne* (Nematoda) [44], *Entamoeba histolytica* (Amoebozoa, suggested telomere role of tRNA and 5S rDNA arrays) [45] and recently sequenced early diverged ciliate Loxodes (Karyorelictea) [46]. Created with BioRender.com.

Building on the identification of TRs in vascular plants (tracheophytes), we expanded our focus to early-diverging taxa within Viridiplantae, including bryophytes, streptophyte algae, green algae, and rhodophytes. Our earlier finding that land plant TRs, similar to those in ciliates, are transcribed by RNA polymerase III under the control of a type-3 promoter suggested a possible common origin of TRs across the Diaphoretickes phylogenetic megagroup. This megagroup includes Archaeplastida (the former supergroup containing plants) and is associated with Ciliophora and Alveolata within the broader TSAR (Telonemia, Rhizaria, Alveolata, and Stramenopila) group (see Figure 1). However, given the extensive diversity of TRs, traditional sequence homology searches proved inadequate for identifying TRs across such a broad evolutionary scale.

To overcome this limitation, we developed an original strategy that, in addition to targeting the expected TR template region (based on known telomere repeats), utilized a conserved feature of TR transcription: the type-3 promoter, specifically its USE. The USE is also present in some snRNA genes (U-RNAs, MRP-RNAs, SRP-RNAs, and SINEs), allowing its identification in genomic data across species. With the increasing availability of genomic and transcriptomic datasets and the known telomere repeat sequences (e.g. [47,48]), we were able to predict corresponding minimal TR template regions [35]. We used the MEME tool [49] to characterize species-specific USE sequences. The co-occurrence of both elements—the USE and the TR template region (within 200 nt downstream of the USE)—enabled the identification of TR candidates. These candidates were further aligned and analyzed using LocaRNA [50], and alignments were used to build covariance models (CMs) in the Infernal tool [51]. CMs incorporate both sequence and secondary structure conservation, which is particularly important for identifying ncRNAs with conserved secondary structures, despite primary sequence variability.

**Figure 2:**
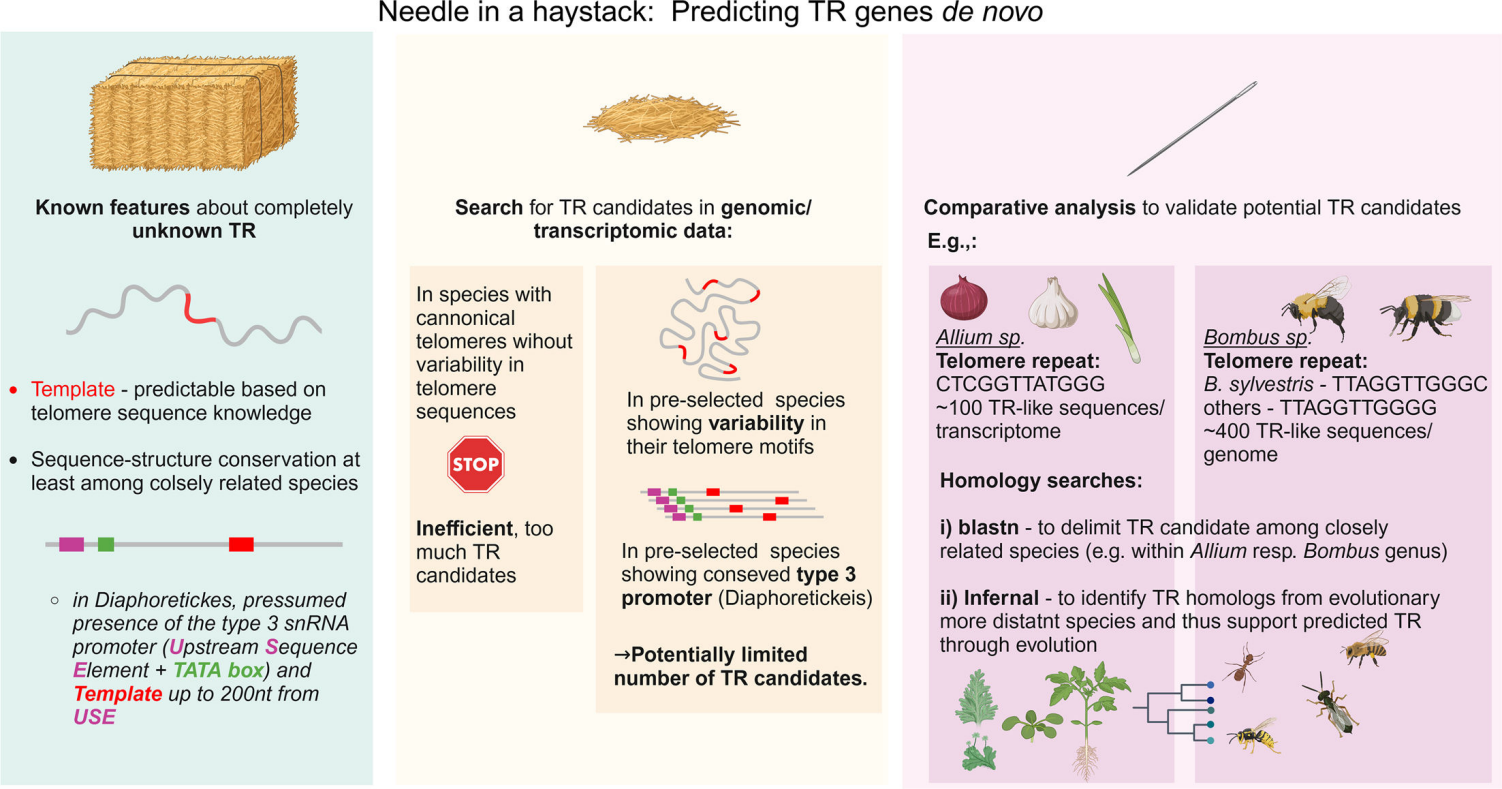
A scheme emphasizing the complexity of identifying TRs in large genomic/transcriptomic datasets, akin to finding a needle in a haystack. This figure presents a conceptual framework for identifying TR genes, highlighting the challenges posed by the vast search space and the elusive nature of TR sequences. The pipeline consists of three key steps: **(i)** Known features of unknown TRs: TR templates can be predicted based on telomere sequence motifs and the conservation of sequence and structure among closely related species. (Note: For TRs in early-diverging land plant and ciliate clades, we presumed the presence of a type 3 snRNA promoter.) **(ii)** Prediction of TR candidates: identifying TR candidates in species with canonical telomeres and no sequence variability is inefficient due to the large number of possible candidates. However, focusing on species with telomere sequence variability can significantly reduce the number of potential TR candidates. **(iii)** Comparative analysis for validation: TR candidates are tested using homology searches, including BLASTN for closely related species and Infernal for distantly related species. Examples from *Allium* and *Bombus* species demonstrate how telomere motif variability facilitates the refinement of TR candidates. Created with BioRender.com.

Using this approach, we identified new TR orthologs in other related species where genomic or transcriptomic data were available [52] (see Figure 2 for a schematic overview). To validate these bioinformatic findings, we conducted transcriptomic analyses, RT-PCR with TR-specific primers, and northern hybridizations using TR-specific probes in representative species. Additionally, TR-knockout mutants were generated in the model moss *Physcomitrium patens*. These mutants exhibited not only a loss of telomerase activity but also the activation of alternative lengthening of telomeres (ALT), leading to the generation of complex tandemly arranged repeats [52]. *In silico* analysis of the newly identified TRs in Diaphoretickes species revealed conserved secondary structure elements, such as pseudoknots and template boundary elements [52]. The secondary structure of *P. patens* TR was further examined experimentally using SHAPE-Map Seq and DMS-Map Seq [53]. The resulting data were analyzed using the recently developed DaVinci single-molecule method [54], which revealed multiple clusters of secondary structures, reflecting the structural dynamics of TR in different functional states. Interestingly, the presumed active (OPEN) conformation of the TR template region constituted only a minor fraction of the total TR under *in vivo* conditions, while the major fraction adopted a CLOSED conformation, possibly corresponding to a catalytically inactive telomerase complex during RNP assembly. The structural polymorphism and dynamic transition between CLOSED and OPEN TR conformations may serve as a regulatory switch for telomerase activity, independent of total TR transcript levels. Notably, these results align with the structure of the TERT–TR complex recently established in *A. thaliana* [55].

In conclusion, our findings provide evidence of a common origin of TRs across the Diaphoretickes phylogenetic megagroup, suggesting that RNA polymerase III-dependent TR transcription is deeply conserved across more than a billion years of evolution.

### Intricate pathways of TR evolution

Earlier studies on telomeres and telomerases in animals (Metazoa, Animalia) suggested a common origin of their TRs, which were classified as H/ACA box snoRNAs transcribed by RNA polymerase II. However, TRs remained unidentified in certain groups within Ecdysozoa, including Arthropods and Nematodes. In a recent study, using the previously described strategy, we identified TRs in the insect orders Hymenoptera and Lepidoptera. Surprisingly, their TRs resemble the type found in plants and across Diaphoretickes (i.e. TRs transcribed by RNA polymerase III under the control of a type-3 promoter), rather than the type expected from their phylogenetic position (Figure 1) and typically observed in other Animalia groups [22]. These findings challenge the current understanding of the monophyletic origin of TRs in animals and suggest an evolutionary shift in TR type and biogenesis that coincided with the divergence of Arthropods. Speculating on the mechanisms driving this evolutionary shift in the Animalia kingdom, we recognize that the evolution of TR is tightly linked to the evolution of its associated cofactors. This dynamic interdependence means that changes in one component necessitate adaptive changes in the other. To maintain the critical roles of telomeres and telomerase in genomic stability, evolution has repurposed pre-existing genetic material. This has facilitated the adaptation or even the neofunctionalization of both protein and RNA components. In the case of Hymenoptera TRs, evolution appears to have co-opted ncRNAs with transcriptional machinery similar to spliceosomal RNAs. Concurrently, these TRs exhibit convergent evolutionary similarities to TRs found in plants and ciliates, likely reflecting structural or functional parallels. Expanding our knowledge of TRs and telomerase components in other Arthropod and Ecdysozoa lineages would provide new insights into the evolution and functional diversity of telomerase systems.

### Changes in telomeric DNA are inherently dangerous, yet they still occur—how is this possible?

**Figure 3:**
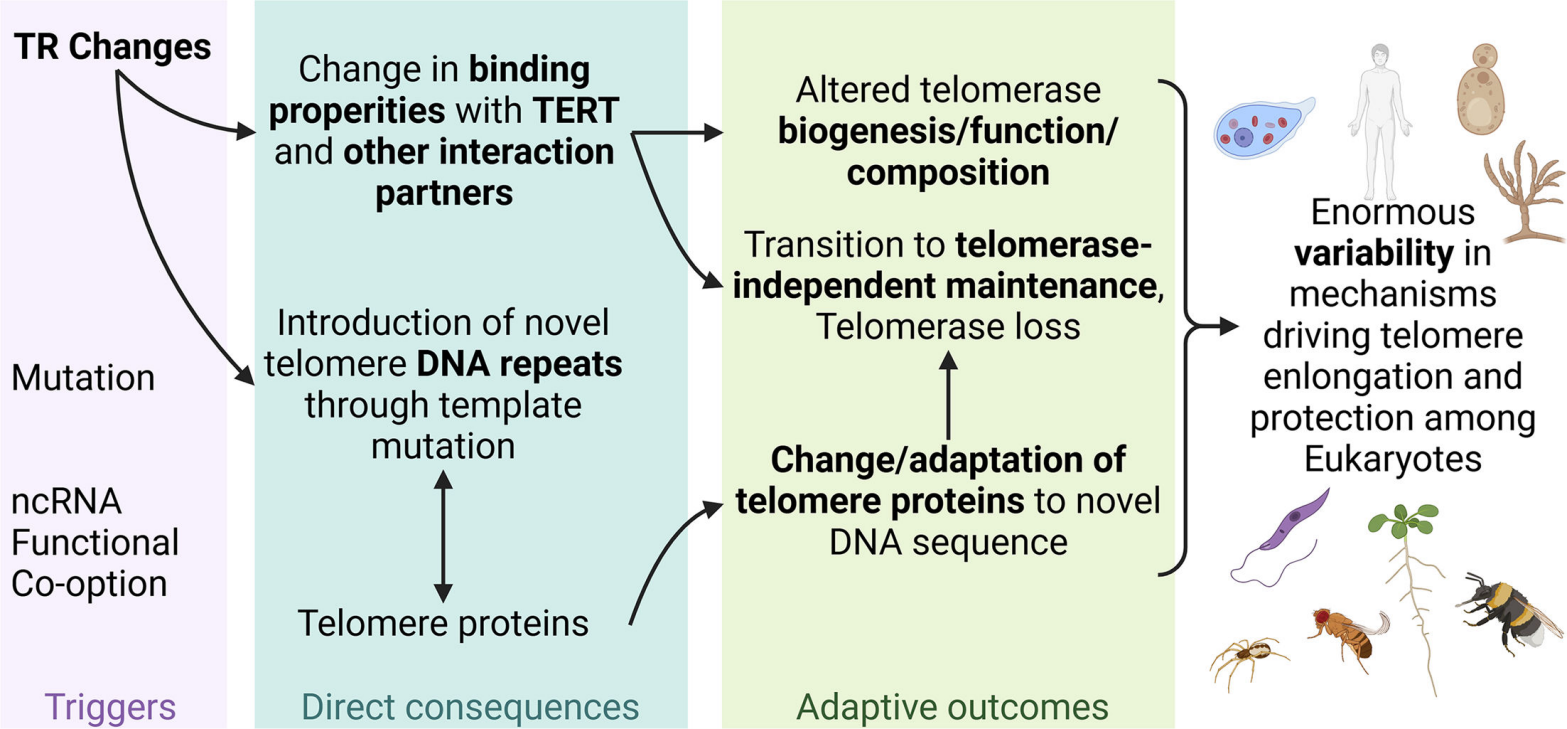
Impact of telomerase RNA variability on telomere/telomerase function. Triggers (purple) such as mutations and functional co-option of non-coding RNAs initiate changes in TR. These result in *direct consequences* (blue) including alterations in binding properties with TERT and other interaction partners, as well as the introduction of novel telomeric DNA repeats through template mutation. These changes drive modifications in telomere-associated proteins to maintain the protective function of telomeres. Over time, adaptive outcomes (green) emerge, such as alterations in telomerase biogenesis, function, and composition, or transitions to telomerase-independent maintenance and telomerase loss. These processes contribute to the enormous variability observed in mechanisms driving telomere elongation and protection across diverse eukaryotic taxa. Created with BioRender.com.

Telomerase, telomeric DNA, and associated proteins form a complex, finely tuned, and highly conserved system that ensures genome integrity by protecting and maintaining chromosome ends. The central component of this telomere maintenance machinery is the TR, which serves as a template for telomere DNA synthesis. Mutations in TR can alter telomeric DNA, disrupting its recognition by telomere-binding proteins. This disruption can lead to the collapse of telomere end-protection and telomerase recruitment functions (see Figure 3). Consequently, changes in telomere and telomerase components can compromise an organism’s viability.

However, molecular innovations in telomere maintenance have occurred multiple times throughout eukaryotic evolution, giving rise to species or taxa with unusual telomeric DNA sequences, novel telomerase components, or even telomerase-independent telomere maintenance mechanisms. This phenomenon has been clearly demonstrated in our previous studies, which detailed the evolutionary transitions in telomeric DNA sequences during plant phylogenesis. These transitions ultimately enabled us to identify TRs that underpin these changes (see above).

To better understand how such changes in TR and telomere structure occur without compromising viability, we decided to systematically investigate these mechanisms. Using a bioinformatic approach developed in our earlier studies, we identified plants harboring TRs with template regions capable of supporting the synthesis of diverse telomeric sequences [56]. Frequently, these TRs were present as two or more paralogs within the examined species. Subsequent experimental analyses showed that, in some cases (e.g. *Vestia foetida* and species of *Capsicum—C. annuum*, *C. baccatum*, and *C. chinense*), these TR paralogs were expressed and involved in telomere synthesis, producing two distinct types of telomeric repeats within the same cell. These species may represent a transitional state—evolution in progress.

In many other species with unusual telomeric sequences, only the TR responsible for templating the ‘unusual’ telomeric repeats has been retained. This could reflect a situation in which the newly synthesized telomeres were compatible with the flexible binding capacity of telomere-associated proteins, or the telomere proteins themselves underwent adaptive evolution during a period of functional redundancy provided by duplicated TRs. Most other plants exhibit only a single copy of TR. However, since we only observe successful evolutionary outcomes, it is difficult to determine the fate of transiently duplicated TR genes, such as those arising from whole-genome or segmental duplications.

We summarize this plausible evolutionary scenario in Figure 3. In this scenario, the initial event in telomere transitions is TR duplication, which introduces functional redundancy without posing an immediate threat to telomere function or organismal viability. Emerging TR paralogs may arise either from local duplication events, resulting in tandem or interspersed TR-duplicated segments lacking synteny, or from large-scale duplications that preserve the synteny of duplicated segments. In our study [56], we identified examples of both types of duplication. Mechanisms underlying local duplications include retrotransposition, transduplication, erroneous DNA repair, replication slippage, and unequal crossover. In contrast, large-scale duplications are often associated with polyploidization, hybridization, or segmental duplication. The critical role of TR paralogs in the evolution of TR genes aligns with the broader concept of redundancy as a driving force in evolutionary processes [57,58]. The presence of redundancy relaxes selective pressure against mutations in the TR template region, allowing potentially harmful mutations in one of the TR copies to be tolerated, thanks to the presence of a functional copy. Simultaneously, this situation provides an opportunity for the organism to ‘test’ the functionality of the mutated allele and possibly adapt to the altered telomeric DNA sequence via the adaptive evolution of telomere-binding proteins or their substitution [59-62]. If the mutated allele proves to be more functional, or if the adaptation process is successful, this may eventually lead to the loss of the original TR allele, although this is not necessarily the outcome.

### Current developments

Current knowledge as well as the latest advancements in telomere and telomerase evolution research have been summarized in a newly established database, *TeloBase* [63]. This database (http://cfb.ceitec.muni.cz/telobase/) addresses the growing gap between contemporary research data and the outdated, fragmented information available in existing resources. *TeloBase* consolidates data on telomere DNA sequences through extensive literature reviews and the analysis of publicly available next-generation sequencing data, creating a comprehensive repository of telomere motif diversity. The database is supplemented by an internal taxonomy that integrates common online taxonomic resources, enabling in-house data filtration and graphical visualization of telomere DNA evolution through heat tree plots.

One notable feature of *TeloBase* is its avoidance of over-reliance on administrators for future data updates. It employs a community-curation system where users can submit new telomere sequences through a simple form, subject to application and approval. This system ensures that the database remains up to date and relevant as the field continues to evolve.

Another challenge we encountered during TR identification was the lack of sufficient bioinformatics expertise among many biologists. As mentioned earlier, TRs are a class of ncRNAs, characterized by low sequence conservation despite relatively high structural conservation across broad phylogenetic ranges. Identifying conserved structural features requires specialized tools such as CMs, which combine sequence alignment with consensus secondary RNA structures. However, running the standard implementation of this approach (e.g. Infernal) requires advanced bioinformatics knowledge, in contrast with more user-friendly tools like BLAST.

This gap is partially addressed by *RNAcentral*, which allows searches for homologs across a wide range of ncRNA sequence collections from diverse organisms. However, it does not support searches directly across genome assemblies. To make the identification of unknown ncRNA homologs, including TRs, more accessible, we developed a tool called *GERONIMO* [64]. This tool enables evolutionary searches across hundreds of genomes in a fully automated manner, presenting results with taxonomic context in summary tables and visualizations for easier analysis.

*GERONIMO* also provides additional information on homologous sequences, including flanking genomic regions, enabling analyses of promoter motifs or gene collinearity, which aids in validating the results. This makes *GERONIMO* a valuable resource for identifying homologs of functionally important ncRNAs, including TRs, across diverse taxonomic groups. By removing the limitations that previously confined ncRNA research to model organisms and their close relatives, *GERONIMO* expands our understanding of ncRNAs in their evolutionary context.

## Concluding remarks

Research into telomere biology and telomerase evolution has made remarkable strides over the last decade. Much of this progress stems from moving beyond traditional model systems and leveraging the explosion of genomic and transcriptomic data available now. The evolution of TR appears far more complex than previously assumed. For instance, while a common origin of TRs has been confirmed in plants, ciliates, and across the broader Diaphoretickes megagroup, the assumption of a shared origin in animals has been challenged by the discovery of ‘plant-type’ TRs in certain insect groups (Figure 1).

These findings represent a significant shift in our understanding of telomerase evolution, underscoring the dynamic nature of this field. It is both exciting and rewarding to witness and contribute to these groundbreaking discoveries as our knowledge of telomere biology continues to evolve.

## Abbreviations

ALT: alternative lengthening of telomeres
AcTR: *Allium cepa* TR
BLAST: Basic Local Alignment Search Tool
CMs: covariance models
H/ACA: H box (consensus ANANNA) and the ACA box (ACA) conserved sequence RNA motifs
POT1a: protection of telomeres 1a, 1b
RNP: ribonucleoprotein
TATA box: promoter region of TATAAA consensus sequence
TBE: template boundary element
TBE: template boundary element
TERT: telomerase reverse transcriptase
TLC1: telomerase component 1
TR: telomerase RNA
TSAR: Telonemia, Rhizaria, Alveolata, and Stramenopila
USE: upstream sequence element of a promoter
USE: upstream sequence element
hTR: human TR
lncRNA: long non-coding RNA
mTR: mouse TR
ncRNA: non-coding RNA
snoRNA: small nucleolar RNAs
